# Evaluation of the Anti-microbial Efficacy of a Novel Endodontic Irrigant Against Enterococcus faecalis: An In Vitro Study

**DOI:** 10.7759/cureus.46410

**Published:** 2023-10-03

**Authors:** Gladson Selvakumar, Abinaya Raveendran, Swathika B, Ganesan S, Prem Kumar E, Gopal Chandra Sanyal

**Affiliations:** 1 Department of Conservative Dentistry and Endodontics, Mahatma Gandhi Postgraduate Institute of Dental Sciences, Puducherry, IND; 2 Department of Pediatric and Preventive Dentistry, Mahatma Gandhi Postgraduate Institute of Dental Sciences, Puducherry, IND

**Keywords:** chlorhexidine, anti-microbial, simvastatin, e. faecalis, irrigant

## Abstract

Introduction:* Enterococcus faecalis* is a constant microbiome that plays an inevitable role in the etiology of peri radicular lesions after endodontic treatment, chronic and, apical periodontitis and, recently, in periimplantitis. The effective biomechanical preparation and the use of potent irrigating solutions will permit bacterial neutralization and toxin inactivation, leading to the success of endodontic treatment. This study aimed to evaluate the "anti-microbial" efficacy of simvastatin (SMV) against *E. *faecalis as an endodontic irrigant.

Materials and methods: In this invitro experimental study, the antimicrobial efficacy of SMV was evaluated against *E. faecalis* using the agar diffusion method. The samples were divided randomly into the following groups. GROUP 1: SMV solution 1 μM/L concentration, GROUP 2: SMV solution 5 μM/L concentration, GROUP 3: SMV solution 10 μM/L concentration, GROUP 4: 2% chlorhexidine gluconate (CHX) solution (positive control), and GROUP 5: normal saline (negative control). Linear measurement was done by measuring the zones of inhibition around the medicaments in the cavities in millimeters. Results were tabulated.

Results: The results of the study have shown the zone of inhibition of Group 4 (2% CHX solution) is 19 mm, which demonstrated the best outcome. When comparing the test samples, Group 3 (SMV solution 10 M/L concentration) has the best zone of inhibition, measuring 14 mm, followed by Group 2 (SMV solution 5 M/L concentration), which is 9 mm.

Conclusion: The results of this in vitro study have proven that SMV's anti-microbial activity, albeit less potent than CHX in this in vitro investigation, has demonstrated that it can be utilized as an efficient endodontic irrigant.

## Introduction

Endodontic infections are multi-microbial although obligatory anaerobic bacteria predominates [1]. Because it is such a tenacious microbe, *Enterococcus *plays a substantial role in the development of periradicular lesions following root canal therapy. Additionally, apical periodontitis, chronic periodontitis, and, most recently, periimplantitis have all been associated with it. Due to biofilm development and the organism's physicochemical characteristics, which enable it to alter the prevailing environmental and nutritional conditions, it can even thrive in constrained environments [2]. *E. faecalis* can colonize inside dentinal tubules to a depth of >1000 μm or close to the cementum [3]. Therefore, effective biomechanical preparation and the use of potent irrigating solutions will permit bacterial neutralization and toxin inactivation, which plays a key role in the success of endodontic treatment [4]. Therefore, irrigation is important because it helps clean root canal system areas that are difficult for devices to reach, which in turn lowers the amount of bacteria.

The number of bacteria in the root canal has been reduced or eliminated using a variety of irrigant treatments in endodontics such as sodium hypochlorite, chlorhexidine gluconate (CHX), tetraclean, MTAD, herbal irrigants, ozone, etc. [5]. An effective endodontic irrigant should be antibacterial, safe for periapical tissues, able to dissolve tissue or debris, lubricate the canal, and facilitate smear layer removal [6].

Sodium hypochlorite has been considered the gold standard for irrigation because of its ability to dissolve organic matter and high antimicrobial potential [7]. However, it has been discovered that the drawbacks of utilizing sodium hypochlorite at maximum concentration as an endodontic irrigant are linked to its cytotoxicity when applied beyond the peri-radicular tissues. Extrusion of it can result in intense pain, instant swelling, and heavy bleeding [6].

CHX is used widely as an endodontic irrigant and medicament. With regard to bacteria, CHX has a wide range of effects, including Gram-positive and Gram-negative bacteria, spores, viruses, yeast, and dermatophytes. It shows increased antimicrobial action toward endodontic organisms such as *Staphylococcus aureus, Porphyromonas endodontalis, Porphyromonas gingivalis, Prevotella intermedia, Enterococcus faecalis, Candida albicans, *and *Streptococcus mutants* [8]. A recent study showed a substantial reduction of CHX effectiveness in the long term because of the electrostatic attraction of CHX to extracellular polymeric substances, limiting CHX penetration and reducing its concentration in deep biofilm layers [9]. However, there is no endodontic irrigant that can fulfill all the ideal requirements. Hence, the search for an endodontic irrigant that can fulfill all the requirements still exists.

Simvastatin (SMV) is a structural analog of HMG-CoA (3-hydroxy-3-methylglutaryl-coenzyme). The medication is regarded as the first-line treatment for hyperlipidemia and has an extended record of use across the globe. It is also a renowned safe and affordable medication. It is a novel medicinal substance that is still undergoing dental research.

It exerts antimicrobial properties as one of its pleiotropic effects. Numerous studies have revealed its antimicrobial efficacy against periodontal pathogens [10,11]. Scientific studies have proven it to be an effective material for vital pulp therapy and as an intracanal medicament [11-15]. Therefore, this study aimed to evaluate the “anti-microbial efficacy of SMV as an endodontic irrigant.”

## Materials and methods

Preparation of SMV solution

**Figure 1:**
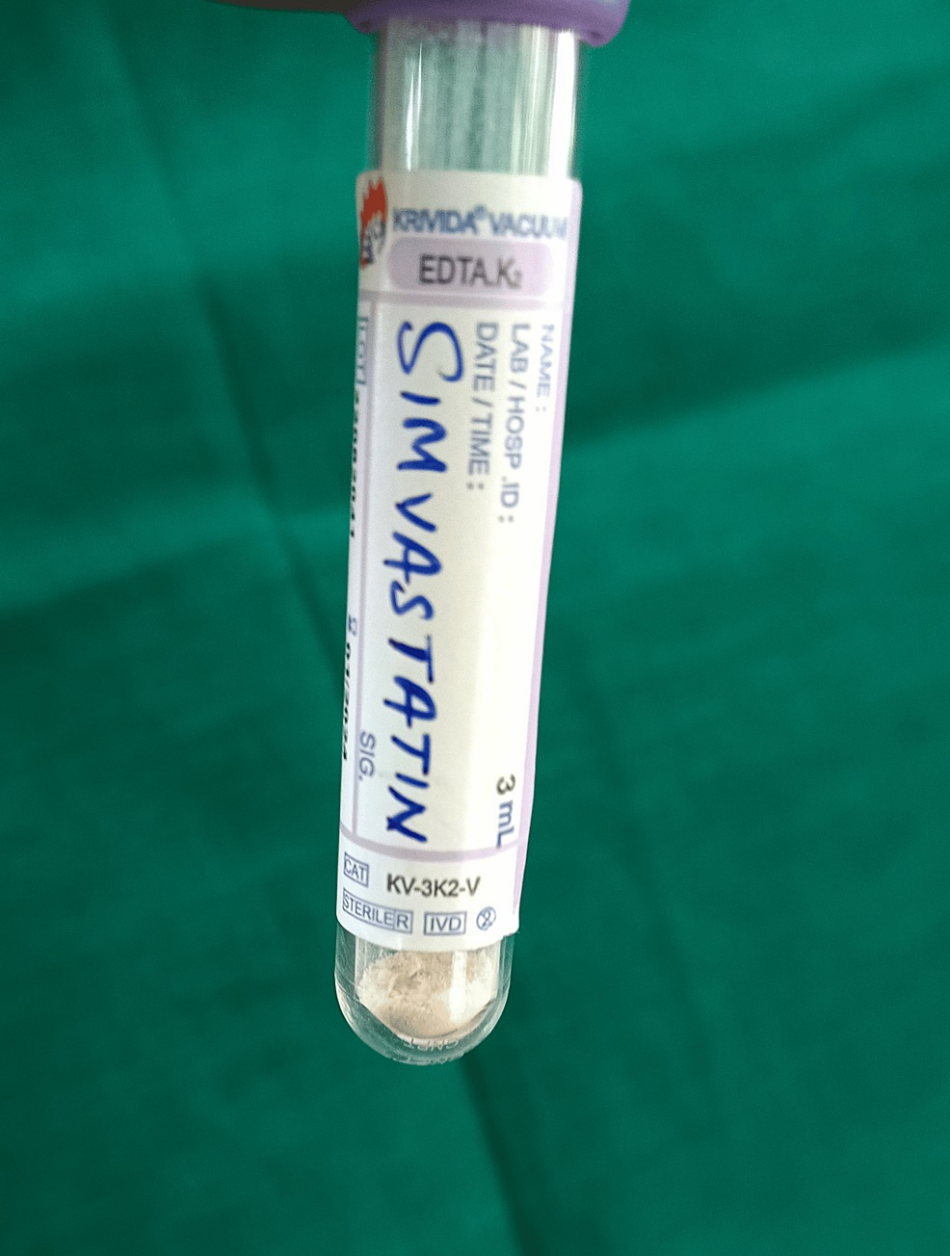
Image of pure simvastatin powder Image of pure simvastatin powder

**Figure 2:**
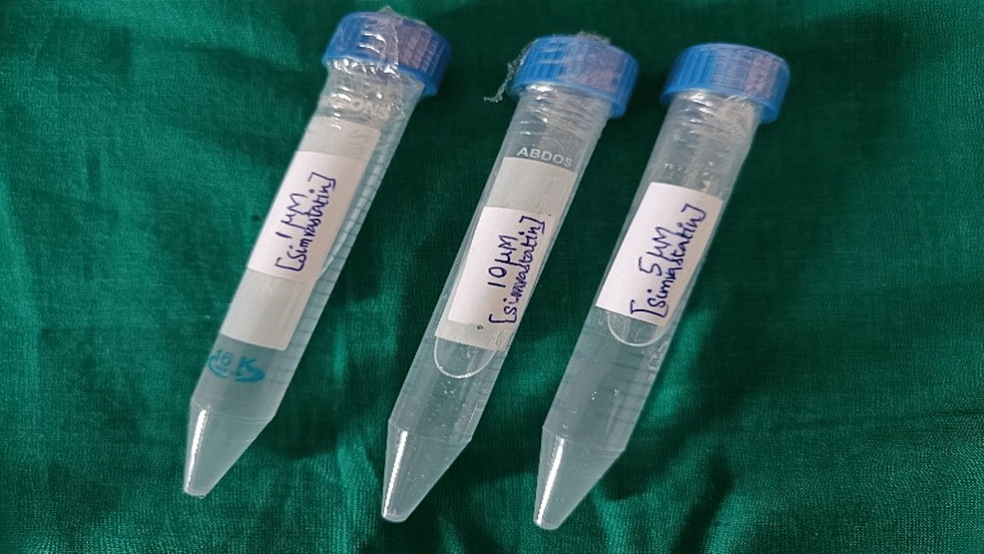
Images of simvastatin solution of 1 μM/L, 5 μM/L, and 10 μM/L Simvastatin powder was combined with distilled water to create three different concentrations of SMV under aseptic conditions.

SMV (Sigma Aldrich Industries Pvt. Ltd., Germany, batch number S6196, CAS number 79902-63-9) was used to obtain pure simvastatin powder (Figure 1). SMV powder was combined with 10 mL of distilled water using a vortex device to create a concentration of 1 M/L of SMV under aseptic conditions. Similar to that, SMV concentrations of 5 M/L and 10 M/L were created (Figure 2). A single-use Eppendorf tube was filled with 0.5 mL of the prepared SMV, which was then stored at 4 °C [12].

Sample division

To evaluate the anti-microbial efficacy the groups were divided as

GROUP 1: SMV solution 1 μM/L concentration

GROUP 2: SMV solution 5 μM/L concentration

GROUP 3: v solution 10 μM/L concentration

GROUP 4: 2% CHX solution (positive control)

GROUP 5: Normal saline (negative control)

Agar diffusion test

**Figure 3:**
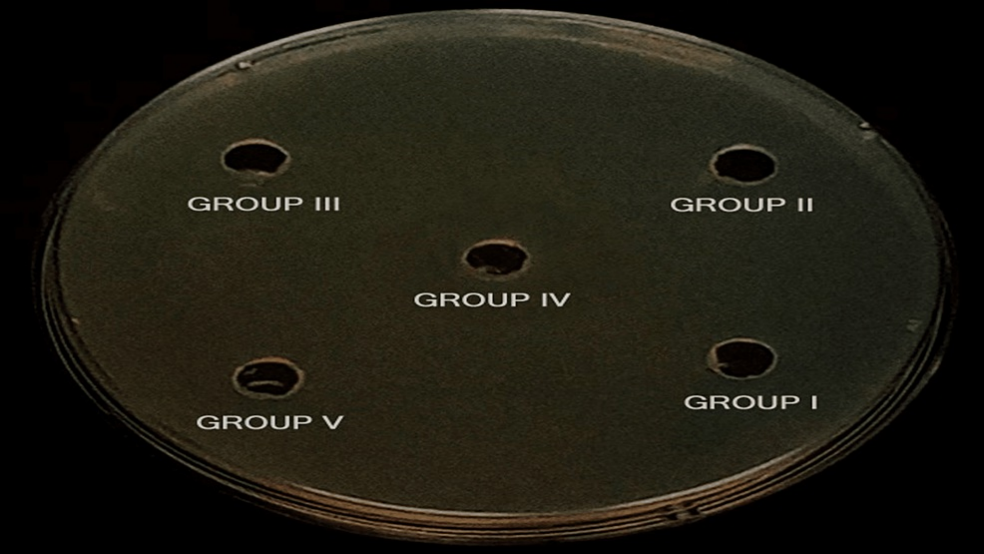
Mueller-Hinton broth Five small wells (6 mm diameter) were dug in the agar and filled with the study medicaments

For this experiment, Mueller-Hinton agar was utilized. Mueller-Hinton agar was used to cultivate a pure culture of *E. faecalis*. The *E. faecalis *strain used in this study was MTCC 2729, which was then inoculated into Mueller-Hinton Broth (HiMedia, Mumbai, India) and incubated at 37 °C overnight. Streaks were then created onto the agar plates (Figure 3).

In a Petri dish, small wells (6 mm diameter) were dug in the agar and filled with the study medicaments. Five cavities were filled with medicaments - the quantity used for each cavity was 5 μL. The Petri dishes were incubated at 37 °C for 48 hours. The study aimed to assess the zone of inhibition using the agar diffusion test. Results were calculated by linear measurement of the zone of inhibition in millimeters [2,16]. The results were tabulated.

## Results

**Figure 4:**
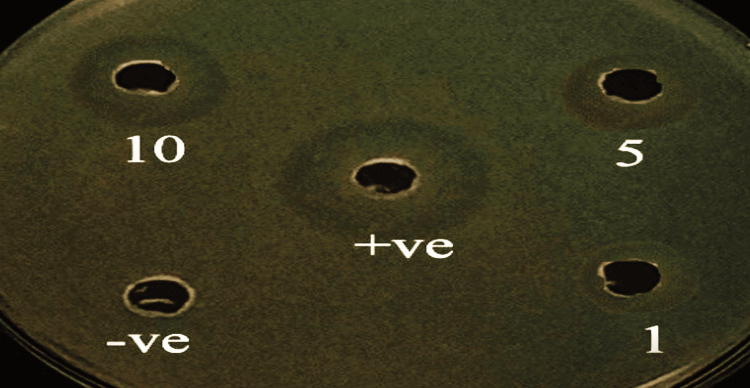
Zone of inhibition in millimeters (mm) GROUP 1 (simvastatin solution 1 μM/L concentration) = 6 mm; GROUP 2 (simvastatin solution 5 μM/L concentration) = 9 mm; GROUP 3 (simvastatin solution 10 μM/L concentration) = 14 mm; GROUP 4 (2% chlorhexidine gluconate solution) = 19 mm; GROUP 5 (normal saline) = 0

The results according to the agar diffusion method have shown that the SMV solution of 10 µM/L concentration has better anti-microbial efficacy among the tested group next to the control group (Table 1). The zone of inhibition of the positive control (2% CHX solution), as shown in (Figure 4), is 19 mm, which demonstrated the best outcome. When comparing the test samples, Group 3 (SVM solution 10 M/L concentration) has the best zone of inhibition, measuring 14 mm, followed by Group 2 (SMV solution 5 M/L concentration), which is 9 mm; Group 1 (SMV solution 1 M/L concentration), which is 6 mm; and Group 5 (negative control), which has the least favorable zone of inhibition, measuring nil. Since we did not have multiple observations for the zone of inhibition, no test of significance was used to compare between the groups.

**Table 1:** Zone of inhibition in millimeters (mm)

Group	Group 1, Simvastatin solution 1 μM/L concentration (mm)	Group 2, Simvastatin solution 5 μM/L concentration (mm)	Group 3, Simvastatin solution 10 μM/L concentration (mm)	Group 4, Positive control (mm)	Group 5, Negative control (mm)
Zone of inhibition	6	9	14	19	0

## Discussion

The results of this study have shown SMV solution 10 μM/L concentration has comparable anti-microbial action against *E. faecalis *similar to CHX.

The key to a successful root canal procedure is irrigation. It performs a number of vital functions, some of which depend on the irrigant employed, including lowering friction between the instrument and the dentine, enhancing the cutting efficiency of the files, dissolving tissue, cooling the file and tooth, and having cleaning and antimicrobial/antibiofilm effects [17]. Additionally, the only means to affect the parts of the root canal wall that are not reached by mechanical instruments is by irrigation.

Because of its high level of resistance to a variety of antimicrobial agents, *E. faecalis*, a facultative anaerobic Gram-positive coccus, has been used in several prior studies on the effectiveness of endodontic irrigants and has been linked to persistent root canal infections [18-21].

Due to its broad-spectrum antibacterial activity and significantly lower toxicity than NaOCl, CHX is a substitute irrigant for NaOCl [22]. With four chlorophenyl rings, two biguanide groups, and a central hexamethylene bridge, CHX is a symmetrical molecule [23]. CHX functions generally as an antiplaque and antigingivitic agent. It combats fungus, viruses, and other diseases. CHX's substantivity, which relates to oral retentiveness, is one of its most distinctive distinguishing characteristics. It relies on a number of variables, including the concentration, pH, temperature, and length of time that solutions are in contact with oral structures [24]. For root canal therapy, a 2% CHX dosage is most frequently utilized. The capacity of root fillings to prevent fluid entry into the root canal system through the apical foramen is unaffected by CHX-treated canals, making it safe to use as an irrigant [25].

CHX's characteristics, such as its water solubility, substantivity, low toxicity, and broad spectrum of antibacterial activity, have also ignited interest in its application in endodontics. Due to the precipitation and/or coagulation of the cytoplasm, most likely brought on by protein cross-linking, CHX exhibits a bacteriostatic effect at low doses and a bactericidal effect at higher ones.

Although CHX satisfies most of the ideal requirements of endodontic irrigants, biofilm or any other organic material cannot be dissolved by it. Permanent root filling is likely to result in residual organic tissue weakening the seal's quality [26].

Statin cholesterol‐lowering drugs are the first choice drugs to control hyperlipidemia. Due to a wide range of health benefits in addition to their cholesterol-lowering properties, statins have recently attracted significant attention as a new treatment strategy for several oral health conditions. SMV has been demonstrated to have a variety of beneficial effects, including those that are antibacterial, anti-inflammatory, immunomodulatory, antioxidant, and bone-forming [11]. Moreover, SMV is cost-effective as compared to other commercially available irrigants [12].

Several studies have shown that SMV has an antibacterial effect against various oral microbial pathogens. Statins prevent the enzyme 3 hydroxy 3 methylglutaryl coenzyme A (HMG CoA) reductase from functioning, which reduces the production of endogenous cholesterol. All higher eukaryotes and many bacteria have the rate-limiting enzyme HMG-CoA reductase, which is essential for the human mevalonate pathway [11]. The HMG CoA reductase gene (mvaE gene) is present in *E. faecalis* (MTCC 2729) that was used in this study.

In a study published by Brilhante et al. in 2015, SMV showed an inhibitory effect against *Candida *spp. and *Cryptococcus *spp. with minimum inhibitory concentration (MIC) values ranging from 15.6 to 1000 mg L^−1^ and from 62.5 to 1000 mg L^−1^, respectively, and it was able to inhibit growth and mature biofilms of *Candida *spp. and *Cryptococcus *spp [27]. In 2017 Whitaker et al. explained the bacteriostatic effect of SMV against selected oral streptococci species found commonly in the oral cavity and stated that SMV inhibits the growth of *Streptococcus mutans, Streptococcus sanguis, Streptococcus anginosus, *and *Streptococcus salivarius*, with MIC values ranging from 7.8 to 15.6 μg/ml [28].

In a case report published by Fawzy et al. in 2020, SMV was used as an intracanal medicament for the management of cracked teeth. SMV's anti-inflammatory, antibacterial, antioxidant, and bone/wound healing characteristics could account for the rapid pain alleviation that followed the application of the substance inside the canal [13].

Fan et al., in 2020, confirmed the enhanced antibacterial activity against *E. faecalis *of Ag⁺ by SMV. The antibacterial efficacy of SMV can be attributed to its potential to Inhibit the synthesis of endogenous cholesterol. It could also reduce the production of extracellular polymeric substances (EPS) by bacteria. Furthermore, SMV was found to be able to reduce the adenosine kinase (ADK) gene expression of microorganisms [29].

Rahimi et al., in 2023, assessed the antimicrobial effects of different concentrations of SMV versus triple antibiotic paste (TAP) on *E. faecalis *biofilms and concluded that SMV has shown effective antimicrobial effects against *E. faecalis* [30]. The results of the study were found to be in favor of the current study.

In this present study, SMV 10 µM/L concentration has shown better anti-microbial efficacy among the studied concentrations next to 2% CHX solution. Thus, considering the results of our study, SMV 10 µM/L concentration can be used as an endodontic irrigant. The findings of this experimental investigation cannot be compared to those of other studies because it is the first of its type to investigate SMV in three different concentrations as a possible endodontic irrigant. However, the use of SMV in the current case is regarded as an empirical treatment that was based on experimental investigations. Additional clinical research should be undertaken to investigate its side effects and mode of action.

## Conclusions

As the study was experimental in nature and outcomes could differ in practical settings, additional clinical studies are required to evaluate the effectiveness of simvastatin at various dosage levels. Within the limitations of the study, it has been determined that SVM's anti-microbial property has been shown to be effective against *E. faecalis*. Simvastatin was tested in this investigation in a variety of concentrations, but the 10 M/L concentration produced the best results overall. Because of its anti-microbial properties and affordability, it can be a reliable substitute for other commercially available irrigants.
